# Rare IFT140-Associated Phenotype of Cranioectodermal Dysplasia and Features of Diagnostic Journey in Patients with Suspected Ciliopathies

**DOI:** 10.3390/genes14081553

**Published:** 2023-07-28

**Authors:** Margarita Sharova, Tatyana Markova, Maria Sumina, Marina Petukhova, Maria Bulakh, Oxana Ryzhkova, Tatyana Nagornova, Sofya Ionova, Andrey Marakhonov, Elena Dadali, Sergey Kutsev

**Affiliations:** 1 Research Centre for Medical Genetics, 115522 Moscow, Russia; 2 State Healthcare Institution of Sverdlovsk Region “Clinical and Diagnostic Center “Mother’s and Child Health Protection”, 620067 Ekaterinburg, Russia

**Keywords:** *IFT140*, cranioectodermal dysplasia, ciliopathy, thoracic dysplasia with short ribs

## Abstract

Here we present a patient with a cranioectodermal phenotype associated with pathogenic variants in the *IFT140* gene. Most frequently, pathogenic variants in *IFT140* correspond to the phenotype of Mainzer–Saldino syndrome. Only four patients have previously been described with this cranioectodermal phenotype and variants in *IFT140*. In comparison to other IFT140-cranioectodermal patients, our proband had similar skeletal features among with early onset end-stage renal failure that required kidney transplantation but did not have common ophthalmological features such as retinopathy, optic nerve atrophy, or nystagmus. Following exome sequencing, a splicing variant and exons 27–30 tandem duplication were suspected and further validated. The two other patients with Mainzer–Saldino syndrome that we described displayed a typical clinical picture but a special diagnostic journey. In both cases, at first only one pathogenic variant was detected following panel or exome NGS sequencing. Further WGS was performed for one of them where tandem duplication was found. Screening the third patient for the same tandem duplication was successful and revealed the presence of this duplication. Thus, we suggest that the description of the clinical feature polymorphism in a rare IFT140-cranioectodermal phenotype is extremely important for providing genetic counseling for families, as well as the formation of the correct diagnostic path for patients with a variant in *IFT140*.

## 1. Introduction

The *IFT140* gene encodes for the IFT140 protein, which is part of a large multi-protein complex called the intraflagellar transport (IFT) complex. This complex is essential for the formation, maintenance, and function of the cilia, which are hair-like structures that extend from the surface of diverse types of cells. Biallelic pathogenic variants in *IFT140* or other IFT-A complex genes can cause defective retrograde cilial transport [1]. This can result in a range of disorders known as ciliopathies, which can affect multiple organ systems and cause a variety of symptoms, including vision and hearing loss, skeletal abnormalities, kidney disease, and developmental delay [2,3].

The *IFT140* gene is associated with several different phenotypes, such as Short-rib thoracic dysplasia, type 9 syndrome (OMIM #266920), and Retinitis pigmentosa, type 80 (OMIM #617781), as per OMIM database. Short-rib thoracic dysplasia, type 9 syndrome encompasses phenotypic spectrum from less severe Mainzer–Saldino syndrome to asphyxiating thoracic dystrophy or Jeune syndrome. Mainzer–Saldino syndrome (MSS) is characterized by a combination of the cone-shaped epiphyses of the phalanges, early onset of renal failure, and retinal dystrophy. Symptoms of thoracic dysplasia are not dominant in the phenotype and patients can develop a narrow chest and recurrent respiratory infections without asphyxia or other severe symptoms. Occasionally, patients can present liver fibrosis, mild developmental delay, and cerebellar ataxia [4,5]. More than 40 variants in the *IFT140* gene have been described in patients with MSS or other short-rib thoracic dysplasia phenotypes, according to the HGMD database to date (2022.1 version).

Additionally, according to recent publications, the *IFT140* gene is associated with another newly described phenotype called Cranioectodermal dysplasia or Sensenbrenner syndrome [6,7,8]. Cranioectodermal dysplasia (CED) is characterized by the combination of MSS symptoms with craniofacial abnormalities (frontal bossing, dolichocephaly, sagittal craniosynostosis, epicanthal folds, telecanthus, hypertelorism) and ectodermal anomalies (sparse and thin hair, dental hypoplasia, oligodontia, nail dysplasia) [9,10]. There are only a few patients described in the literature with CED associated with the IFT140 gene [6,7,8].

Here, we present the fifth patient with *IFT140* CED phenotype. We compared this patient with two other MSS patients with IFT140 variants in our cohort and their diagnostic journeys.

## 2. Materials and Methods

### 2.1. Subjects

Three probands aged 2, 3, and 7 years along with their parents underwent physical examination and genetic investigation at the Research Centre for Medical Genetics, Moscow. Written informed consents were obtained from the families. The study was performed in accordance with the Declaration of Helsinki and approved by the Institutional Review Board of the Research Centre for Medical Genetics, Moscow, Russia.

### 2.2. Genetic Analysis

Blood samples from probands and unaffected parents were collected, and genomic DNA was extracted using standard methods. 

Targeted panel sequencing of 166 genes responsible for the development of hereditary skeletal pathology was performed to clarify the diagnosis for two patients. New generation sequencing was carried out using Ion Torrent S5 sequencer with an average depth of at least 80×, covering ≥90–94% of targeted areas. To annotate the identified variants, standard HGVS nomenclature was used. Sequencing data were processed using a standard Ion Torrent automated algorithm. To assess the population frequencies of identified variants, samples from the «1000 Genome» projects, ESP6500, and The Genome Aggregation Database v2.1.1 were used. To assess clinical significance of the identified variants, OMIM and the HGMD databases were used. A list of analyzed genes is available in the Supplementary Materials, Figure S1.

Clinical exome sequencing was performed for the identification of mutations in the proband’s DNA. Target enrichment with a SeqCap EZ HyperCap Workflow solution capture array (Roche Sequencing Solutions, Pleasanton, CA, USA) included coding the regions of 6640 genes currently described as clinically significant in OMIM and the Human Gene Mutation Database (HGMD). Paired-end sequencing (2 × 75 b.p.) was carried out on an Illumina NextSeq 500. The coding sequence of IFT140 gene was completely covered with coverage varying from 10× up to 600×. Sequencing data were processed using a standard computer-based algorithm from Illumina and Basespace software (Enrichment 3.1.0). Mapped reads were visualized with Integrative Genomics Viewer (IGV) software (© 2013–2018 Broad Institute, and the Regents of the University of California, Pleasanton, CA, USA). The variant-filtering algorithm was based on frequencies of less than 1% in the Genome Aggregation Database (gnomAD v.2.1.1) and coding region sequence effects, such as missense, nonsense, coding indels, and splice sites. The variants’ clinical significance was evaluated according to the massive parallel sequencing (MPS) data interpretation guidelines [11,12]. The cDNA and protein positions in IFT140 corresponded to transcript NM_014714.4.

Whole-genome sequencing was performed using a DNBSEQ-G400 instrument in pair-ended mode (2 × 150 b.p.) with an average on-target coverage of 30× with MGIEasy FS PCR-Free DNA Library Prep Set (BGI, Beijing, China) for library preparation (Genomed Ltd., Moscow, Russia). Bioinformatic analysis was performed using an in-house software pipeline as described earlier (PMID: 29504900) with modifications. In brief, it included quality control of raw reads (FastQC tool v.0.11.5), followed by read mapping to the hg19 human genome assembly (minimap2 v.2.24-r1122), the sorting of the alignments, and the marking of duplicates (Picard Toolkit v.2.18.14). Base recalibration and variant calling were performed using GATK3.8. Variant annotation was performed using the Annovar tool (v.2018Apr16). CNV and SV analysis was performed using the Manta tool (v.1.6.0). Further filtering was performed through functional consequences and population frequencies, according to the ACMG recommendations as well as clinical relevance determined by the Human Phenotype Ontology database [13].

The validation of a ~5 kb duplication in the IFT140 gene and the analysis of segregation in the family were performed using a test system based on multiplex PCR. The test system included several oligonucleotide primers: the first pair (IFT140-Fint30/IFT140-Rint30) was located in the intron 30 of the IFT140 gene and flanked the left boundary of the duplicated fragment and the second pair (ITF140-Fint27/IFT140-Rex27) was located within the duplicated region closer to its right edge in the exon 27 area of the *IFT140* gene. In the case of the wild-type allele, the IFT140-Fint30/IFT140-Rint30 primer pair amplifies a 420 base pair fragment and the ITF140-Fint27/IFT140-Rex27 primer pair amplifies a 143 base pair fragment. In the presence of a direct tandem duplication, an additional 1004 base pair fragment appears among the amplification products, which corresponds to the area of junction between the left and right copies of the duplication (Supplementary Figure S1). This fragment was then analyzed via Sanger sequencing to confirm the results of NGS sequencing and to determine the breakpoints of the duplication formation.

## 3. Results

### 3.1. Family 1

**Figure 1:**
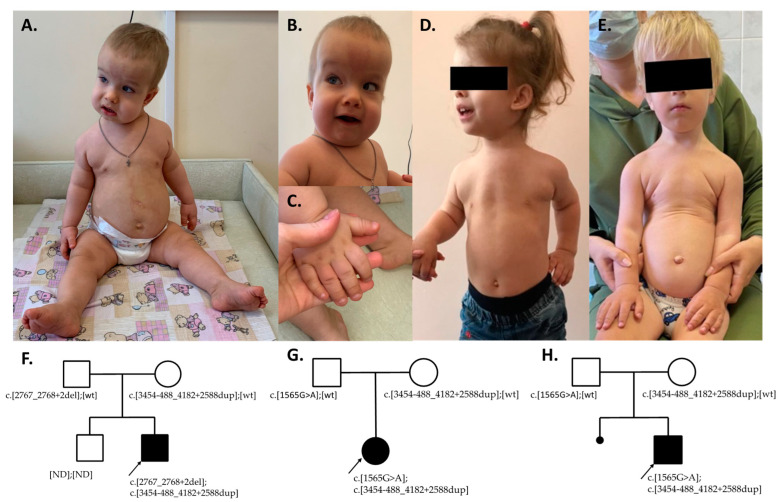
Phenotypes of three patients in our cohort. Patient 1 (**A**–**C**), Patient 2 (**D**), and Patient 3 (**E**). Patient’s 1 skeletal features such as narrow chest, rhizomelic arm and leg shortening, and dolichocephaly are shown in (**A**). Patient 1′s dysmorphic facial features, such as frontal bossing, strabismus, and epicanthus are shown in (**B**), and the nail abnormalities and brachycephaly are shown in (**C**). The skeletal abnormalities of Patients 2 and 3 (narrow chest, rhizomelic arm shortening) are shown in (**D**,**E**), respectively. (**F**–**H**) The pedigrees of the three patients with their respective genotypes. ND—genotype unknown.

Proband 1 was born at 40 weeks of gestation from a seventh pregnancy in a non-consanguineous family with a history of five miscarriages and one healthy older brother. Delivery was normal with an APGAR score of 8/9, a birth weight of 4200 g, and a length of 55 cm. The proband reached early motor milestones with a delay—he started to hold his head at 2 months and sat unsupported at 19 months. Parents noticed a change in the shape of the child’s skull at the age of 6 months, and the isolated craniosynostosis of the sagittal suture was then diagnosed. Cranial vault remodeling surgery was performed at the age of 7 months. During the preparation for the surgery, high levels of protein in the child’s urine were detected. At 8 months, the patient already had stage 4 chronic kidney disease (CKD). At 9 months, he had CKD 5D stage and started dialysis. At 20 months, the patient underwent nephrectomy and kidney transplantation. The boy underwent physical examination at our center at the age of 23 months. According to the examination, he had dolichocephaly, short neck, rhizomelic extremity shortening, narrow chest, and brachydactyly. Other notable dysmorphic features included frontal bossing, the light nevus flammeus of the forehead, epicanthus, strabismus, full cheeks, midface hypoplasia, coarse fair hair (never cut), and thin and short nails (Figure 1A–C). Motor milestones were reached with a delay, as the proband could not stand without support. Ciliopathy with a cranioectodermal dysplasia was suspected due to a combination of renal failure and craniosynostosis with ectodermal features. Detailed phenotype analysis at the time of examination is presented in Table 1. 

Through the results of exome sequencing, a heterozygous deletion at the exon 21-intron 21 border of the IFT140 gene (chr16:1575885CACTA>C) affecting the canonical splicing donor site NM_014714.4: c.2767_2768+2del was identified. The variant was previously described in two patients [8]. According to segregation analysis, the variant was inherited from the father. Also, based on the analysis of coverage, the presence of a heterozygous duplication of exons 27–31 of the *IFT140* was suspected (Supplementary Figure S2). Using multiplex PCR, a duplication containing the region from exon 27 to exon 30 confirmed the duplication in the proband’s and the mother’s sample. The duplication was also previously described as pathogenic NM_014714.4:c.3454-488_4182+2588dup (p.Tyr1152_Thr1394dup) [14].

### 3.2. Family 2

Proband 2 was referred to our center at the age of 28 months for the first time. The girl was delivered at 38 weeks of gestation from a first pregnancy in a non-consanguineous family. She was delivered through the C-section with hypoxia and a APGAR score of 5/7, a birth weight of 3640 g, and a length of 55 cm. She also reached early motor milestones with a delay, as she started holding her head up at 2 months and walking at 19 months. She was diagnosed with a nystagmus and the partial atrophy of the optic nerve in the first month of her life. Chest deformation, chronic bronchitis, and an increased level of protein in the urine also was noted in the second year of life, as well as rhizomelic limb shortening and brachydactyly with the same X-ray findings. CKD stage 3 was diagnosed at the age of 4 years. The proband presented with end-stage chronic kidney disease at the age of 7 years, requiring dialysis treatment. Phenotype features shown in Figure 1D.

NGS panel sequencing was performed, and only one previously described pathogenic variant—NM_014714.4:c.1565G>A (p.Gly522Glu)—was identified. The second variant was not found during analysis. In order to find the second variant in the *IFT140* gene, WGS was performed. As for proband 1, tandem duplication with borders 1565635_1571303 was suspected and validated via multiplex PCR. Both variants were found in a compound heterozygous state.

### 3.3. Family 3

The last patient in our cohort (Proband 3) was born at 40 weeks of gestation from a second pregnancy in a non-consanguineous family. He was delivered via C-section because of cord entanglement and fetus hypoxia and had an APGAR score of 6/7, a birth weight of 4040 g, and a length of 53 cm. Similarly, proband 3 reached early motor milestones with a delay: he started holding his head up at 2 months, sitting unsupported at 12 months, and started walking at 16 months. He was diagnosed with nystagmus, hypermetropy, and the partial atrophy of the optic nerve during the first few months of his life. In the second year of his life, he was found to have a chest deformation, a frequent prolonged cough, and was then diagnosed with chronic bronchitis. Later that year, an increased level of protein in his urine was detected. At 36 months, he was found to have CKD 3. An X-ray examination found the cone-shaped epiphyses of the metacarpal bones and phalanges. He underwent physical examination at our center at the age of 37 months. The boy had a rhizomelic shortening of the upper and lower limbs, a narrow chest, brachydactyly, generalized joint laxity, and a wide umbilical ring (Figure 1E). He also had horizontal–rotary nystagmus and could not fully focus on objects.

Exome sequencing were performed as a first line of diagnosis. The same previously described pathogenic variant—c.1565G>A (p.Gly522Glu)—that was inherited from the father was found. The second variant was not found during the analysis. We decided to screen for the 27–30 duplication using primers for the intron 31–intron 26 junction and were able to successfully find it in both cases. We validated the duplication using multiplex PCR in the probands’ and the mothers’ samples.

## 4. Discussion

Pathogenic variants in *IFT140* are associated with various phenotypes of ciliopathies. The most frequent phenotype is MSS syndrome. The rarest phenotype of IFT140-associated ciliopathy is cranioectodermal dysplasia, which is frequently referred to as Sensenbrenner syndrome. To date, five types of CED, associated with *WDR35*, *IFT122*, *WDR19*, *IFT43*, and *IFT52* genes that form the IFT transport complex, have been described [15]. Only four patients with symptoms similar to CED manifestations have previously been described [6,7,8] with variants in the *IFT140* gene. The presence of skeletal and kidney symptoms are common for all patients with pathogenic variants in the *IFT140*. However, patients with CED compared to MSS most likely showed craniosynostosis, mild ectodermal abnormalities, and earlier end-stage renal failure that required kidney transplantation in early childhood.

Our Patient 1 is the fifth described patient with the CED phenotype and *IFT140* variants. All described patients had common skeletal abnormalities (narrow chest, rhizomelic limb shortening, brachydactyly) and dysmorphic features (dolichocephaly, high forehead, epicanthus) along with nail, hair, and teeth abnormalities. Similarly to the previously described patients (Table 1), he required kidney replacement therapy at the first year of life—at 9 months old. However, Patient 1 did not have symptoms of retinopathy or other ophthalmologic symptoms except strabismus at the age of two years. Other described CED patients had nystagmus, strabismus, and hyperopia at the ages of 7 months and 3.5 years [6]. Nystagmus, retinal dystrophy, and optic nerve atrophy was also described in a CED patient with the same genotype at the age of 3 months [8]. 

Patients 2 and 3 within our cohort exhibited the characteristic clinical manifestations of the MSS phenotype. However, their molecular diagnostic journey deserves notable consideration. Using these examples, we emphasize the importance of tandem duplication screening in patients with clinically suspected *IFT140*-associated dysplasia when only one genetic variant was detected in the *IFT140* using NGS methods. This simple step allowed us to make the final molecular–genetic diagnosis for two patients in our cohort at once.

**Figure 2:**
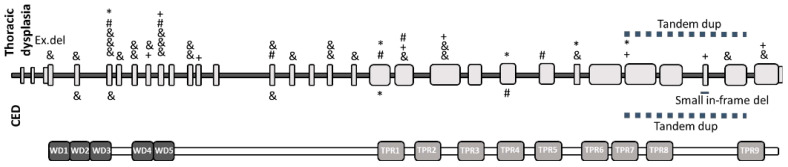
(**Top part**): distribution of previously described variants in the *IFT140* gene contributing to MSS and Juene phenotype above the exon structure scheme and contributing to CED phenotype below the exon structure scheme. *—nonsense variants; #—splicing sites and other intronic variants; &—missense variants; + frameshift variants. (**Bottom part**): schematic protein structure correlated to exons with WD and TRP domains.

Additionally, we conducted an analysis of genotype–phenotype correlations among patients described in the literature with the dominant symptoms of thoracic dysplasia and patients described with cranioectodermal dysplasia. The described pathogenic variants are evenly distributed throughout the gene. However, for patients with thoracic dysplasia, a greater number of genetic variants found at the N-terminus are predominantly missense, with slightly fewer genetic variants described in the TPR domain regions (Figure 2).

We did not identify any correlations between the type of variant and phenotype. Moreover, the same genetic variants in a compound heterozygous state led to different phenotypes, as we demonstrated when comparing our patients with those described in the literature. Thus, the presence of phenotypic heterogeneity in the *IFT140* gene cannot be explained by different variant effects on mRNA or protein structure, as previously shown for other genes in skeletal ciliopathies [16,17] or some other genes [18,19]. The abundance of LoF variants in the spectrum of thoracic dysplasia suggests that the complete loss of function of the corresponding protein may be a mechanism of pathogenicity; however, the fact that the same variants can give rise to different phenotypes suggests other possible mechanisms for the development of different phenotypes.

This complicates the genetic counseling of such patients and is a cornerstone in predicting the course of the disease for each individual patient. Perhaps, such phenotypic heterogeneity may be associated with variants in other genes that affect the complex mechanism of ciliary transport that was shown by Walczak-Sztulpa et al. [10]; however, we were not able to conduct a similar study in the three other patients, highlighting the need for further investigation.

Additionally, we believe that in the presence of such allelic heterogeneity, the division of syndromes into Mainzer–Saldino, Jeune, Sensenbrenner, and others is not justified, which is confirmed by one patient [7]. The patient had all the phenotypic features of cranioectodermal dysplasia, as well as recurrent respiratory infections as a manifestation of thoracic dysplasia. Therefore, we can expect not only different phenotypes with the same genotype, like Patient 1 from our article and previously described P1 and P2 [8], or Patients 2 and 3 [6], but also overlapping leading symptoms typical for both thoracic dysplasia and cranioectodermal dysplasia in one patient. We believe that it would be more appropriate to use the terms IFT140-associated ciliopathy or IFT140-associated dysplasia.

## 5. Conclusions

Thus, we believe that the description of our patients and the analysis of genotype-phenotype correlations are extremely important for providing genetic counseling for families with *IFT140*, as well as for establishing the correct diagnostic search route for patients with a variant in *IFT140*.

## Figures and Tables

**Table 1:** Clinical and genetic characterization of the three probands described in the article and previously described patients with the CED phenotype.

Individual	Proband 1 (This Article)	Proband 2 (This Article)	Proband 3 (This Article)	P1 (Walczak-Sztulpa 2022) [8]	P2 (Walczak- Sztulpa 2022) [8]	P1 (Walczak-Sztulpa 2020) [6]	P2 (Walczak-Sztulpa 2020) [6]	P1 Bayat 2017 [7]
Sex	Male	Female	Male	Male	Male	Male	Male	Male
Age of onset	6 months	1 month	2 months	ND	ND	Early neonatal	7 months	3 months
Variant 1	c.2767_2768+2del	c.1565G>A	c.1565G>A	c.2767_2768+2del	c.2767_2768+2del	c.396C>T	c.1565G>A	c.634G>A;
Variant 2	c.3454-488_4182+2588dup	c.3454-488_4182+2588dup	c.3454-488_4182+2588dup	c.3454-488_4182+2588dup	c.3454-488_4182+2588dup	c.3454-488_4182+2588dup	c.3454-488_4182+2588dup	c.2278C>T
Phenotype	CED	MSS	MSS	MSS	CED	CED	CED	MSS/CED
First symptoms	Craniosynostosis, proteinuria	Nystagmus, partial atrophy of the optic nerve	Nystagmus, partial atrophy of the optic nerve	ND	ND	Proteinuria, hematuria	Proteinuria, hematuria	Recurrent respiratory infections
Short stature	+	-	-	+	+	+	ND	+
SD for growth	−2.35	−1.95	−1.56	ND	ND	−3	ND	−4
Short-rib dysplasia	+/−	+	+	+	+	+	+	+
Chronic respiratory infection	-	+	+	+	-	+	+	+
Rhizomelic limb shortening	+	+	+	+	+	+	+	+
Brachydactyly	+	+	+	+	+	+	+	+
Cone-shaped epiphyses	ND	+	+	+	+	ND	Flattened epiphyses	+
Onset of renal involvement	6 months	2 years	2 years	ND	ND	Early neonatal	7 months	ND
Onset of renal replacement therapy	9 months	7 years	-	3 years	9 months	4.5 years	8 months	20 months
Craniosynostosis	+	-	-	+	-	-	-	+
Dysmorphic features	Epicanthus, dolichocephaly, frontal bossing, High forehead, nail abnormalities	-	-	Dolichocephaly, micrognathia, thin hair, malformed widely spaced teeth	Epicanthus, frontal bossing, high forehead, malformed widely spaced teeth, nail abnormalities	Dolichocephaly, high forehead, thin hair, full cheeks, low set prominent ears, long philtrum, microretrognathia	Dolichocephaly, high prominent forehead, “senile-like” face, thin sparse hair, full cheeks, small teeth	Epicanthus, frontal bossing, tall forehead, hypertelorism, wide mouth, small square-shaped teeth, sparse eyebrows, eyelashes
Pigment retinitis	-	+	+	+	+	-	-	+
Other ophthalmic features	Strabismus	Nystagmus	Nystagmus	Nystagmus, optic nerve atrophy (partial)	Nystagmus, optic nerve atrophy	Strabismus, nystagmus, high hyperopia	Hyperopia, nystagmus	Nystagmus, retinal dystrophy

## Data Availability

Not applicable.

## Supplementary Materials

The following supporting information can be downloaded at: https://www.mdpi.com/article/10.3390/genes14081553/s1. Supplementary Materials: List of genes included in targeted panel sequencing; Figure S1: Schematic description of multiplex PCR assay. Two pairs of oligonucleotide primers were used: the first pair (IFT140-Fint30/IFT140-Rint30) was located in intron 30 of the IFT140 gene and flanked the left border of the duplicated fragment, the second pair (ITF140-Fint27/IFT140-Rex27) was located inside the duplicated region closer to its right exon 27 of the IFT140 gene. In the case of the wild-type allele, the IFT140-Fint30/IFT140-Rint30 primer pair amplifies a 420 bp fragment, while the ITF140-Fint27/IFT140-Rex27 pair amplifies a 143 bp fragment. In the presence of the tandem duplication, an additional 1004 bp fragment appears among the amplification products, which corresponds to the junction of the left and right copies of the duplication. This fragment was then analyzed by Sanger sequencing to identify breakpoints in the formation of the duplication; Figure S2: exome coverage analysis. Coverage in a region containing 27–31 exons is 1,5 times higher for proband 1 than in three other unrelated controls (C1-C3) from the same exome performance.
